# Supplementary material for: Knockout of sws2a and sws2b in Medaka (Oryzias latipes) Reveals Their Roles in Regulating Vision-Guided Behavior and Eye Development
Source: Int J Mol Sci. 2023 May 15;24(10):8786. doi: 10.3390/ijms24108786 (PMC10218681; doi:10.3390/ijms24108786)
Supplement: Supplementary file 1 [file ijms-24-08786-s001.zip › ijms-2339900-supplementary.pdf]

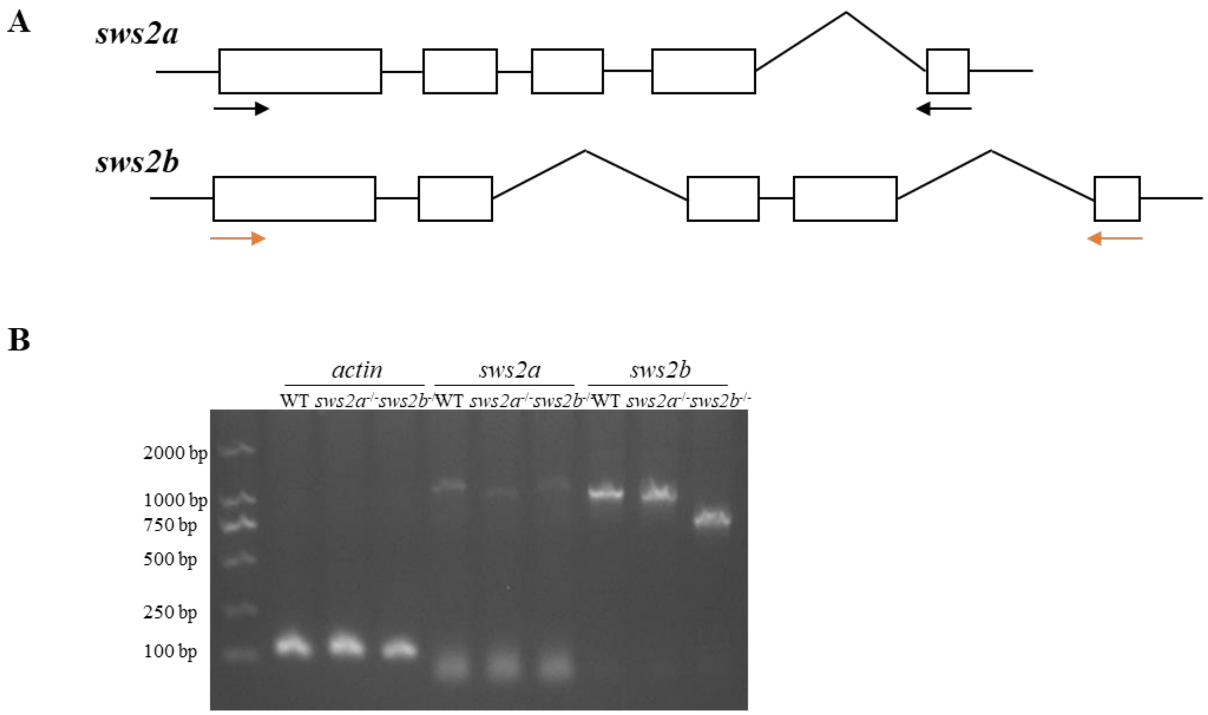

Figure S1. The *sws2a* and *sws2b* mRNA levels were detected in 3-month-old wild-type and *sws2a*$^{-/-}$ and *sws2b*$^{-/-}$ mutant by qRT-PCR. (A) Location of the two primer pairs used for qRT-PCR experiments. (B) The stability of *sws2a* and *sws2b* mRNA are not affected by the mutation.

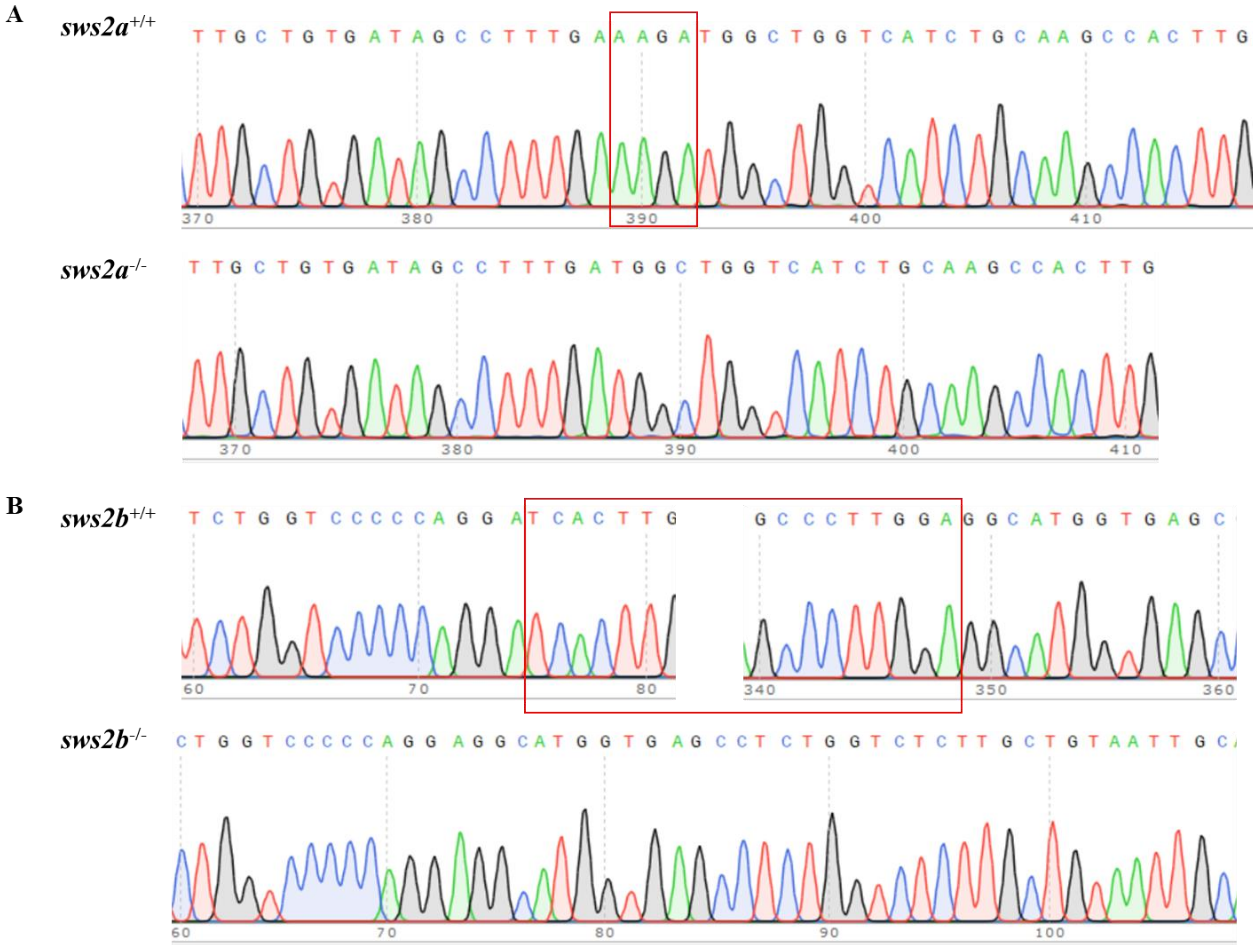

Figure S2. Sequencing the PCR product (spanning the 4-bp (A) and 274-bp (B) deletions) amplified from the cDNA of WT, *sws2a*$^{-/-}$ and *sws2b*$^{-/-}$ mutant medaka retinas. The mutations are present in the mature *sws2a* and *sws2b* mRNA.

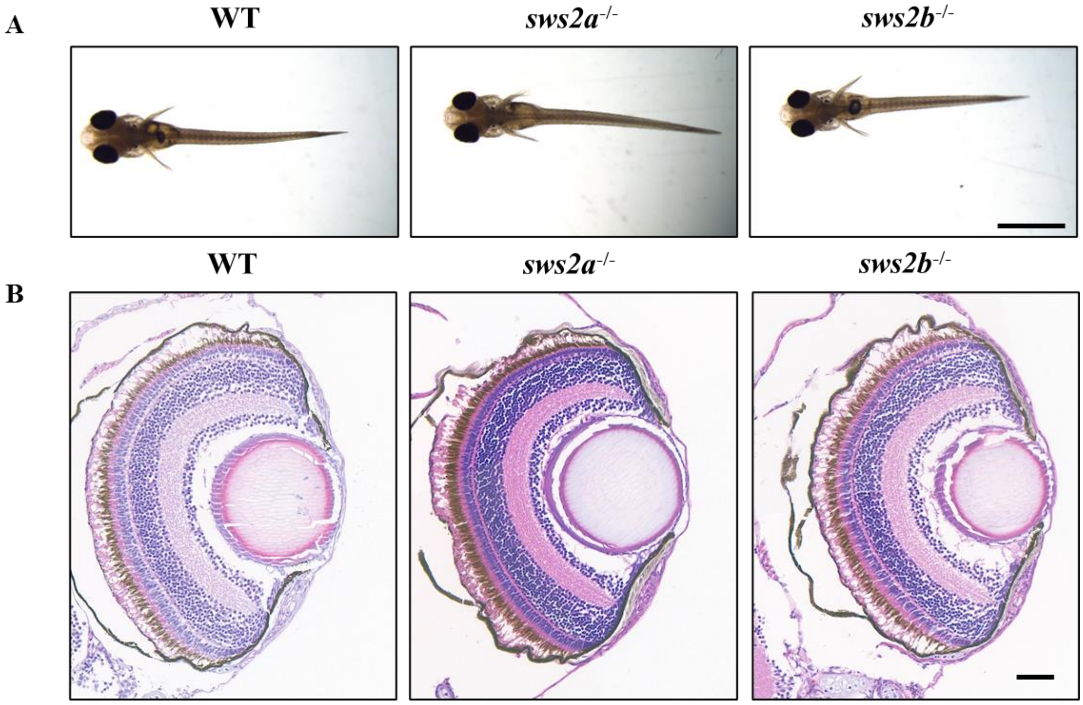

Figure S3. (A) The morphology of WT, *sws2a*$^{-/-}$ and *sws2b*$^{-/-}$ in developmental stages of 6dph. Scale bars indicate 1000 μm. (B) Histological morphology of the retina with H&E staining. Scale bar: 40 μm.

Table S1 The primers used for CRISPR/Cas9.

Gene	Sequence (5'-3')
sws2a guide RNA	GATGACCAGCCATCTTTCAAAGG
sws2a test-F	GGGAGCTGCCAGATGACTTC
sws2a test-R	TCTGTTTACCTGCTCCATCCG
sws2a total length-F	ATGAGGCTCATCAGTGGTGG
sws2a total length-R	CTAAGCTGGTCCGACTTTAG
sws2b guide RNA1	TGGGGCTTCCCAAGTGATCCTGG
sws2b guide RNA2	GAAGGCTTTACTGCAGCCCTTGG
sws2b test-F	AGTGAGGAGCATCAAATATTGTCC
sws2b test-R	CTACTCCATCCCACCAGAGGT
sws2b total length-F	ATGAGGGGAAATCGTCTTGT
sws2b total length-R	TTAGGAAGGGCCGACTTTTG

Table S2 The primers for *q*RT-PCR.

Gene	Gene No.	Forward (5'-3')	Reverse (5'-3')
actin	NM_001104808.1	TATCATTCGCCTGAAACCGAT	CTTTGCACATGCCAGATCCG
sws2a	XM_004069227.4	TGTTACCTGGGTGTGTGCTC	GAACCGCGAAGCAGAAACAG
sws2b	NM_001104654.1	TTCTGCTTTGCCGTCCCTT	AAGCAACAACCATGACGACCA
gdf6a	XM_004086629.4	TTCCGTTCATCCAAATCCGC	CCTTAATTCTGCCCCGACCA
foxq2	XM_011473845.3	ATCCAGAGAAATCCGCCGAC	TAAGTTGTGGCGAACGCTGT
sws1	NM_001104656.1	ACAAAGACTACCGGCTCGTCA	GCTTCCTCCATCTTCTTTCCGA
rh2-a	NM_001104655.1	TTTGGTTGGCTGGTCAAGGT	CACAAGGCTGCCATAGGTGA
rh2-b	XM_004086130.4	TGCACTTCTTCGTCCCAGTC	CCAAGCTACCAGGAAGCCAA
rh2-c	XM_004086132.4	CTTCTGTGCCGTTGAGGGAT	ACAAGCCAGAGCCATTACCC
lws	XM_004069094.4	TGAGGGCTATGTGGTCTCCA	ACCTGCTCCATCCAAAGACG
gnat2	XM_004070401.3	GTGAGTCGGGAAAAAGCACC	CCAGAGCCAGAACCGAAGTC
arr3b	XM_020706457.2	TATCTTGCCTGTGCCTTCCG	GAGCATGGGAGATTGAGGGG
rho	NM_001104695.1	CTCGTTACATCCCAGAGGGC	ATGATGACAACCATGCGGGT
gnat1	XM_004084384.4	AAGCCCATCTGAGCATGTGT	TGATGATGTCGGTTACGGCA
gnb3a	XM_004077988.4	CAGCCTCAAAGACCAGATTACGG	TCATCTGCACACGTCCCAC
gnb3b	NM_001104851.1	GAAAGCTGAAATGGATGCACTGAA	ACATGCTCCCATCATTTGCTG
grk7a	NM_001104661.1	CCACCAAGGACGTCATCCA	CGTGTTTCCTCGGATCCTC
grk7b	XM_004075815.3	TGAGAGGCAGAAGATTACTGACA	GCACAACACAAACCTCACCAAA
pde6c	XM_004080270.3	CGGCAAACTCCAAAAAGCCA	TGAGGGGGAAGGTGAGCATA
six3a	NM_001122928.1	GACCCGTATCCAAACCCCAG	GCTCGGTCCTATTGCTTGGT
six3b	NM_001104760.1	CCCCGACTCAAGTAGGGAAC	GGAACGTGTCTCCATCTGCC
six6	XM_004082283.4	GGCTACTGGACTTACACCCAC	TTTGCTGGACAAACTGGCCT
six7	XM_004079929.4	TACCGCATCCGCAAGAAGTT	GGTTCGGGTACGGATCTTGG
pax6a	XM_004082283.4	GCCAGTGACAAGCAGCAAAT	TTCACCGAGATGCCGTTCTC
pax6b	XM_020714754.2	GCTCAACGGTCAGACAGGAA	CGTCTCCTCTGAATCCTCGC
